# An Absorption Mitigation Technique for Received Signal Strength-Based Target Localization in Underwater Wireless Sensor Networks

**DOI:** 10.3390/s20174698

**Published:** 2020-08-20

**Authors:** Xiaojun Mei, Huafeng Wu, Nasir Saeed, Teng Ma, Jiangfeng Xian, Yanzhen Chen

**Affiliations:** 1 Merchant Marine College, Shanghai Maritime University, Shanghai 201306, China; meixiaojun19@stu.shmtu.edu.cn (X.M.); 201530110005@stu.shmtu.edu.cn (J.X.); chenyanzhen@stu.shmtu.edu.cn (Y.C.); 2 Institute for Systems and Robotics, Instituto Superior Técnico, University of Lisbon, 1049-001 Lisbon, Portugal; mateng_heu@hrbeu.edu.cn; 3 Computer, Electrical, Mathematical Sciences & Engineering Division, King Abdullah University of Science and Technology, Thuwal 23955, Saudi Arabia; mr.nasir.saeed@ieee.org; 4 Science and Technology on Underwater Vehicle Laboratory, Harbin Engineering University, Harbin 150001, China; 5 Department of Electronics and Telecommunications, Politecnico di Torino, 10129 Torino, Italy

**Keywords:** received signal strength (RSS), localization, min–max strategy, robust localization algorithm, absorption mitigation technique (AMT), underwater wireless sensor networks (UWSNs)

## Abstract

Localization is an indispensable technology for underwater wireless sensor networks (UWSNs). In what concerns UWSNs, the accurate location information is not only the requirement of the marine field applications but also the basis of the other corresponding research, for instance, network routing and topology control. Recently, an astonishing surge of interest has been drawn in the received signal strength (RSS)-based scheme due to cost-effectiveness and synchronization-free compared with others. However, unlike the terrestrial wireless sensor networks (WSNs), the acoustic signal may suffer the absorption loss in the underwater environment besides the path loss, which degrades the localization accuracy and limits the capability of the RSS-based technology in UWSNs. In this context, a robust localization method with an absorption mitigation technique (AMT) is developed. First, an RSS-based analytically tractable measurement model is conducted, where the maximum likelihood estimator (MLE) is derived. Nevertheless, it is quite challenging to solve the problem using MLE under a non-convex expression. Therefore, by exploiting certain approximations, the considered localization problem is converted into an optimization expression with a maximum absorption loss involved. A min–max strategy is then presented, with which the problem is turned to minimize the worst situation of the absorption loss. After a simple manipulation, the problem is further investigated as a generalized trust region sub-problem (GTRS) framework. Although the GTRS is a non-convex scheme, the solution can be obtained through an iteration method by introducing a multiplier. In addition, the closed-form expression of the Cramer–Rao lower bound (CRLB) of the analytically tractable measurement model is derived. Numerical simulations demonstrate the effectiveness of the proposed method compared with the state-of-the-art approaches in different scenarios.

## 1. Introduction

**Figure 1:**
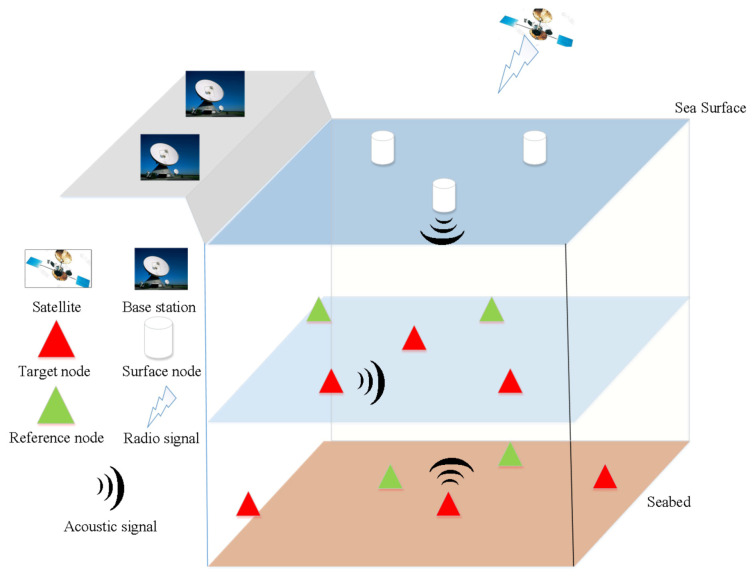
System architecture of underwater wireless sensor networks (UWSNs).

The ocean is vast, covering 140 million square miles, some 72 percent of the Earth’s surface, one of the most valuable natural resources that attract people to explore [1]. As an efficient tool for exploration, underwater wireless sensor networks (UWSNs) have played a pivotal role in a vast number of scientific or commercial missions in civilian and military applications [2,3,4,5,6,7]. It is worth noting that the data collected by sensors are meaningful only when the latter are geo-referenced [8]. However, it is infeasible to obtain the location information because traditional localization methods that rely on GPS techniques cannot be utilized in the underwater environment [9]. In this context, the message interaction, usually using acoustic communications, between surface nodes (or other reference nodes with known locations) and the submerged target must be carried out for localization [10]. The general system architecture of UWSNs is shown in Figure 1. With the knowledge of some internode distances (ranges), the targets’ position would be determined, and thus a considerable body of research has been investigated in the literature regarding localization in UWSNs [4,6,7,8,10,11,12,13,14,15,16,17,18,19,20,21,22,23,24,25,26].

Two main categories could be concluded among the techniques, i.e., the range-based scheme and the range-free scheme [16]. In the range-based scheme, the position is estimated by the measurements through some specific ranging methods, including time of arrival (TOA), time difference of arrival (TDOA), angle of arrival (AOA), and received signal strength (RSS) [7,10]. On the contrary, the estimate procedure could be done through, for instance, hop counts or sensors density, in the range-free scheme without any measurements involved. Although no extra facilities need to be pre-installed, the range-free scheme only can provide a rough estimate [16]. To satisfy a certain level of quality for localization in some tasks, the range-based scheme seems to be a better choice and has been widely used in marine applications [10,16,23].

Regarding the range-based scheme, the RSS-based method has been drawn much attention in the studies due to cost-effectiveness and synchronization-free, compared with TOA, AOA, and TDOA [26]. However, unlike terrestrial wireless sensor networks (WSNs), it is quite challenging to utilize the RSS-based method to locate the target in UWSNs because the acoustic signal may suffer the absorption loss in the underwater environment besides the path loss [15,27]. The hybrid attenuation of the signal may dramatically degrade localization accuracy and limit the RSS-based technology capability in UWSNs. For this reason, researchers have explored various estimators to improve the localization accuracy under such signal loss [4,6,11,13,14,19,22,24,28,29,30].

To name a few, the authors in [4] have presented an RSS-based localization framework for energy harvesting UWSNs, which reduces the localization error of the shortest path for each block kernel matrix by using a majorization-approach-based localization method (MABL). MABL can accurately locate the target under a good quality of the initial guess with relatively low energy consumption. However, as an iterative method via first-order Taylor linearization approximation, the localization accuracy of MABL may not be guaranteed if the quality of the initial guess is terrible. In this case, the authors in [24] have proposed an initial guess-free method, which converted the considered localization problem into a generalized trust-region subproblem (GTRS). A novel weighted least squares (NWLS) has been developed with a known transmit power scheme (NWLS-K) and an unknown transmit power scheme (NWLS-U). The same transformation strategy has been developed in [11], different from [24], the authors transformed the original problem into a mixed semidefinite programming/second-order cone programming (SD/SOCP) problem for reaching an efficient solution. However, both NWLS in [24] and SD/SOCP in [11] were investigated under the low transmission frequency with a relatively small absorption loss. When it comes to a relatively significant absorption loss, the localization accuracy of NWLS and SD/SOCP cannot be guaranteed. In [14], the authors have proposed a robust, non-cooperative localization algorithm (RNLA), wherein a robust function is developed. The three-dimensional localization problem has been transformed into a two-dimensional localization problem in [14] with prior knowledge of the sea depth. Although RNLA can reduce the estimation error in the presence of outliers, the authors did not consider the signal loss caused by the absorption effect. In addition, the authors in [19] have investigated the localization error caused by inhomogeneous underwater medium and presented an oversampled matched filter-based RSS localization method (OSMF-RSS) under a low transmission frequency. The localization error caused by the inhomogeneous transmission is mitigated by using OSMF-RSS. Unfortunately, OSMF-RSS is based on the Gauss–Newton method, which needs a good start-point in the iteration. If the start-point is far from the exact point, the method could get lost. In other words, OSMF-RSS is infeasible to obtain a global solution.

While considerable efforts have been devoted to improving the localization accuracy in terms of the RSS-based scheme, the general effectiveness of these techniques has remained elusive in practice. Most existing works were investigated at a relatively low transmission frequency, for instance, 10 kHz to 100 kHz in [11,13,24], which, in other words, means a relatively low transmission data rate [26]. If one would like to have a higher transmission data rate, it must level up the transmission frequency. However, the problem is that the absorption loss would increase dramatically over the rise in the transmission frequency, according to [15]. For instance, if the distance between a receiver and a transmitter is 200 m while the transmission frequency is 484 kHz, i.e., the absorption coefficient is 0.1 dB/m [13,15], the absorption loss would reach 20 dB, which could degrade the localization accuracy dramatically. In this case, from the practical point of view, it is entirely meaningful to investigate an absorption mitigation technique to decrease the adverse impact of the absorption on localization in UWSNs.

In this context, a robust localization method, i.e., absorption mitigation technique (AMT), is presented. The considered localization problem is transformed into an optimization expression by exploiting certain approximations. With a maximum absorption loss introduced, the problem is divided into two subproblems by exploiting a min–max strategy. Moreover, after a simple manipulation, the optimization problem is further investigated in a GTRS framework, wherein a bisection method with a multiplier is deployed to figure out the solution. In addition, to mimic the situation of the dynamics in the presence of currents, we execute the simulations in which all sensors (anchors and the target) are deployed randomly at each Monte Carlo trial (MCT). In other words, the positions of all sensors in the area of interest are not fixed. To the best of our knowledge, such this absorption mitigation method for localization in such dynamic UWSNs has not been fully addressed.

The main contributions of the paper are summarized as follows:

(1) A min–max strategy is presented, where we minimize the worst situation of the absorption loss with the prior knowledge of the area of interest. The adverse impact of absorption loss on localization is mitigated through iteration.
(2) The considered localization problem is converted into an optimization expression by exploiting certain approximations and further solved in a GTRS framework.
(3) A closed-form expression of Cramer–Rao Low Bound (CRLB) in terms of the considered RSS-based analytically tractable measurement model is conducted.

To organize the paper, we formulate the problem in Section 2. In Section 3, the proposed method, AMT, is illustrated. Comprehensive simulation results are discussed in Section 4. In the last section, Section 5, we conclude this paper.

## 2. Problem Formulation

Consider a 3-Dimensional UWSN containing N reference/anchor nodes with known locations and a target whose position needs to be determined. Suppose the position of the $i^{\mathrm{th}}$ anchor node at time t is $a_{i}={\left[a_{ix},a_{iy},a_{iz}\right]}^{T}$, where i=1,…,N and the target’s position is $x={\left[x_{1},x_{2},x_{3}\right]}^{T}$. We assume that the target could transmit the acoustic signal with RSS information to anchors, of which the signal is modeled as [11,24]

(1) $$P_{ri}=P_{0}-10\alpha \log_{10}\frac{\left\|x-a_{i}\right\|}{d_{0}}-\alpha_{f}\left\|x-a_{i}\right\|+\eta_{i},$$

where $P_{ri}$ denotes the received signal power of the $i^{\mathrm{th}}$ anchor node from the target, $P_{0}$ is the transmit power of the target, α represents the path loss exponent, ‖⋅‖ is the $\ell_{2}$ norm, $d_{0}$ is the reference distance (1 m), $\eta_{i}$ is the shadowing noise modeled as Gaussian distribution with zero mean and variance $\sigma_{i}^{2}$, and $\alpha_{f}$ is the absorption coefficient that can be obtained from Thorp’s formula with a frequency f following [15]

(2) $$\alpha_{f}=0.11\frac{f^{2}}{1+f^{2}}+44\frac{f^{2}}{4100+f^{2}}+2.75\times 10^{-4}f^{2}+0.003.$$

**Figure 2:**
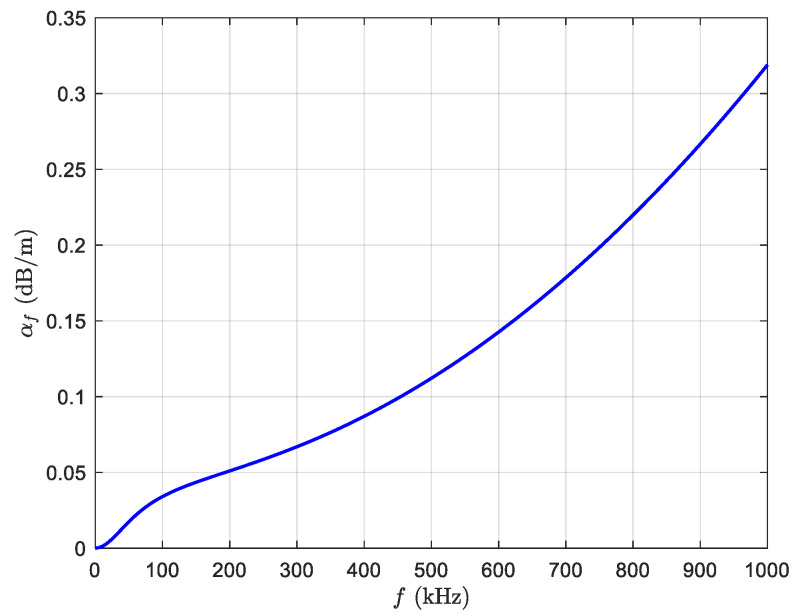
Relationship of the frequency and the absorption coefficient.

The relationship between f and $\alpha_{f}$ is shown in Figure 2, according to [15]. In addition, it should be noted that a standard RSS-based terrestrial localization scheme is obtained if $\alpha_{f}=0$.

Let $c_{i}=\alpha_{f}\left\|x-a_{i}\right\|$, then (1) can be rewritten as

(3) $$P_{ri}=P_{0}-c_{i}-10\alpha \log_{10}\frac{\left\|x-a_{i}\right\|}{d_{0}}+\eta_{i}.$$

Given the observation vector $P={\left[P_{ri}\right]}^{T}$, the probability density function (PDF) is given as

(4) $$p\left(P|x,c\right)=\prod_{i=1}^{N}\frac{1}{\sqrt{2\pi \sigma_{i}^{2}}}\exp\left\{\frac{{\left(P_{ri}-P_{0}+c_{i}+10\alpha \log_{10}\frac{\left\|x-a_{i}\right\|}{d_{0}}\right)}^{2}}{2\sigma_{i}^{2}}\right\}.$$

By maximizing the joint PDF, the maximum likelihood (ML) estimator will be derived as

(5) $$\hat{x}=\arg\min\sum_{i=1}^{N}\frac{{\left(P_{ri}-P_{0}+c_{i}+10\alpha \log_{10}\frac{\left\|x-a_{i}\right\|}{d_{0}}\right)}^{2}}{2\sigma_{i}^{2}}.$$

However, it is quite challenging to solve the problem in (5) accurately due to its high non-convexity in the presence of $c_{i}$, which is the motivation to develop the proposed method.

## 3. Proposed Method (AMT)

### 3.1. Min–Max Strategy

First, we manipulate a simple transformation from (3) as

(6) $$10^{\frac{P_{ri}}{10\alpha }}\cdot \left\|x-a_{i}\right\|=10^{\frac{P_{0}-c_{i}}{10\alpha }}\cdot 10^{\frac{\eta_{i}}{10\alpha }}.$$

When the noise is relatively small, the right side of (6) can be approximated using the first-order Taylor series expansion as [13,14,24]

(7) $$\mu_{i}\cdot \left\|x-a_{i}\right\|\approx \nu_{i}\cdot (1+\frac{\ln10}{10\alpha }\sigma_{i})=\nu_{i}+\varepsilon_{i},$$

where $\mu_{i}=10^{\frac{P_{ri}}{10\alpha }}$, $v_{i}=10^{\frac{P_{0}-c_{i}}{10\alpha }}$, and $\varepsilon_{i}=v_{i}\cdot \frac{\ln10}{10\alpha }\sigma_{i}$.

**Figure 3:**
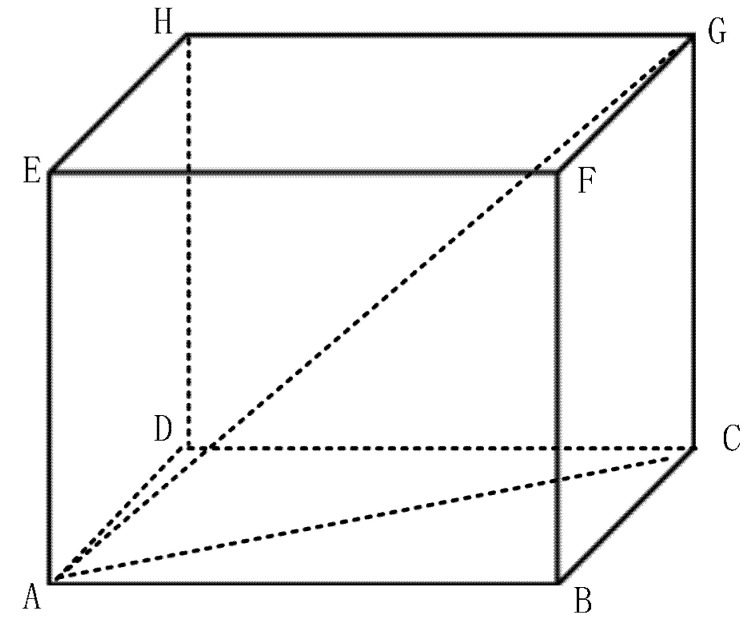
Deployment area of UWSNs with the side length of 100 m.

Further, assuming that the maximum absorption is $c_{\max}$, i.e., $c_{\max}>c_{i}$. Then, the maximum absorption can be determined if the deployment area of UWSNs is known. For instance, if the area is 100 m × 100 m × 100 m, referred to as Figure 3, the maximum distance of two nodes is AG or BH or CE or DF, i.e., 100$\sqrt{5}$ m. Assuming that f is 454 kHz, i.e., $\alpha_{f}=0.1\;\mathrm{dB}/m$ according to [13,15], then $c_{\max}=100\sqrt{5}\cdot \alpha_{f}=10\sqrt{5}\;\mathrm{dB}$.

We insert $\frac{c_{\max}}{2}$ into both sides of (3), then (7) can be expressed as

(8) $${\tilde{\mu }}_{i}\cdot \left\|x-a_{i}\right\|\approx {\tilde{\nu }}_{i}+{\tilde{\varepsilon }}_{i},$$

where ${\tilde{\mu }}_{i}=10^{\frac{P_{ri}+\frac{c_{\max}}{2}}{10\alpha }}$, ${\tilde{v}}_{i}=10^{\frac{P_{0}-c_{i}+\frac{c_{\max}}{2}}{10\alpha }}$, and ${\tilde{\varepsilon }}_{i}={\tilde{v}}_{i}\cdot \frac{\ln10}{10\alpha }\sigma_{i}$.

The original problem in (5) could be rewritten as (9) after squaring.

(9) $$\underset{x}{\arg\min}\sum_{i=1}^{N}{\left({\tilde{\mu }}_{i}^{2}\cdot {\left\|x-a_{i}\right\|}^{2}-{\tilde{\nu }}_{i}^{2}\right)}^{2}.$$

To ensure the objective in (9) is minimum, we should minimize the worst situation, namely,

(10) $$\underset{x}{\mathrm{minimize}}\left\{\underset{{\tilde{\nu }}_{i}}{\mathrm{maximize}}\sum_{i=1}^{N}{\left({\tilde{\mu }}_{i}^{2}\cdot {\left\|x-a_{i}\right\|}^{2}-{\tilde{\nu }}_{i}^{2}\right)}^{2}\right\}.$$

Let ρ(⋅) be the function of ${\tilde{\nu }}_{i}$. The maximization of the function ρ(⋅) subject to ${\tilde{\nu }}_{i}$ can be expressed as $\underset{{\tilde{\nu }}_{i}}{\mathrm{maximize}}\;\sum_{i=1}^{N}\rho^{2}\left({\tilde{\nu }}_{i}\right)$, where $\rho \left({\tilde{\nu }}_{i}\right)=\left|{\tilde{\mu }}_{i}^{2}\cdot {\left\|x-a_{i}\right\|}^{2}-{\tilde{\nu }}_{i}^{2}\right|$. Maximizing the sum of the function is equivalent to maximizing each item of the sum. Thus, (10) can be converted into

(11) $$\sum_{i=1}^{N}{\left[\underset{{\tilde{\nu }}_{i}}{\mathrm{maximize}}\;\rho \left({\tilde{\nu }}_{i}\right)\right]}^{2}=\sum_{i=1}^{N}{\left[\underset{{\tilde{\nu }}_{i}}{\mathrm{maximize}}\;\left|{\tilde{\mu }}_{i}^{2}\cdot {\left\|x-a_{i}\right\|}^{2}-{\tilde{\nu }}_{i}^{2}\right|\right]}^{2}.$$

**Proposition** **1.** *Assume* ${\tilde{v}}_{i1}=10^{\frac{P_{0}+\frac{c_{\max}}{2}}{10\alpha }}$ *and* ${\tilde{v}}_{i2}=10^{\frac{P_{0}-\frac{c_{\max}}{2}}{10\alpha }}$ *, the maximization of* $\rho \left({\tilde{v}}_{i}\right)$ *in (11) has two possible solutions (either* $\rho \left(-\frac{c_{\max}}{2}\right)$ *or* $\rho \left(\frac{c_{\max}}{2}\right)$ *), i.e.,*

(12) $$\underset{{\tilde{\nu }}_{i}}{\mathrm{maximize}}\;\rho \left({\tilde{\nu }}_{i}\right)=\{\begin{array}{c} \rho \left(-\frac{c_{\max}}{2}\right),\mathrm{if}\;A\geq B,\;\\ \rho \left(\frac{c_{\max}}{2}\right),\mathrm{if}\;A<B \end{array},$$

*where* $A=\left|{\tilde{\mu }}_{i}^{2}\cdot {\left\|x-a_{i}\right\|}^{2}-{\tilde{v}}_{i1}^{2}\right|$ *and* $B=\left|{\tilde{\mu }}_{i}^{2}\cdot {\left\|x-a_{i}\right\|}^{2}-{\tilde{v}}_{i2}^{2}\right|$ *.*

**Proof.**  Let ${\tilde{c}}_{i}=c_{i}-\frac{c_{\max}}{2}$, then ${\tilde{v}}_{i}=10^{\frac{P_{0}-{\tilde{c}}_{i}}{10\alpha }}$. According to $c_{\max}>c_{i}$, we have $\left|{\tilde{c}}_{i}\right|=\left|c_{i}-\frac{c_{\max}}{2}\right|\leq \frac{c_{\max}}{2}$. If ${\tilde{c}}_{i}>0$, the extreme value of ${\tilde{c}}_{i}$ can be $\frac{c_{\max}}{2}$, whereas, if ${\tilde{c}}_{i}<0$, the extreme value of ${\tilde{c}}_{i}$ is $-\frac{c_{\max}}{2}$. Therefore, ${\tilde{v}}_{i}$ can be either ${\tilde{v}}_{i1}=10^{\frac{P_{0}+\frac{c_{\max}}{2}}{10\alpha }}$ or ${\tilde{v}}_{i2}=10^{\frac{P_{0}-\frac{c_{\max}}{2}}{10\alpha }}$. In addition, if A>B, the maximization of $\rho \left({\tilde{v}}_{i}\right)$ can be $\rho \left({\tilde{v}}_{i1}\right)$, i.e., $\rho \left(-\frac{c_{\max}}{2}\right)$. Otherwise, the maximization of $\rho \left({\tilde{v}}_{i}\right)$ is $\rho \left({\tilde{v}}_{i2}\right)$, i.e., $\rho \left(\frac{c_{\max}}{2}\right)$. □

It should be noted that max{d,e}≤d+e, for d,e≥0. In this case, by joining the two branches, we convert the problem in (9) into (13) via minimizing an upper bound of $\rho \left({\tilde{v}}_{i}\right)$, i.e.,

(13) $$\underset{x}{\mathrm{minimize}}\;J_{1}+J_{2},$$

where $J_{1}=\sum_{i=1}^{N}{\left({\tilde{\mu }}_{i}^{2}\cdot {\left\|x-a_{i}\right\|}^{2}-{\tilde{\nu }}_{i1}^{2}\right)}^{2}$ and $J_{2}=\sum_{i=1}^{N}{\left({\tilde{\mu }}_{i}^{2}\cdot {\left\|x-a_{i}\right\|}^{2}-{\tilde{\nu }}_{i2}^{2}\right)}^{2}$.

### 3.2. Generalized Trust Region Subproblem (GTRS)

By expanding the squared norm part in (13), the problem is further converted into a GTRS.

(14) $$\begin{array}{l} J\left(y\right)=\underset{y}{\mathrm{minimize}}\;{\left\|\omega (\tilde{℘}y-\tilde{\kappa })\right\|}^{2},\\ \;\mathrm{subject}\;\mathrm{to}\;y^{T}Dy+2f^{T}y=0, \end{array}$$

where $y={\left[x^{T},{\left\|x\right\|}^{2}\right]}^{T}$, $\omega =\mathrm{diag}\left(1_{2N}\right)$, $\tilde{℘}=\left[℘\;;℘\right]$, $\tilde{\kappa }=\left[\kappa_{1};\kappa_{2}\right]$, and

(15) $$℘=\left[\begin{array}{cc} \begin{array}{l} -2{\tilde{\mu }}_{1}{}^{2}a_{1}^{T}\\ \vdots \\ -2{\tilde{\mu }}_{N}{}^{2}a_{N}^{T} \end{array} & \begin{array}{l} {\tilde{\mu }}_{1}{}^{2}\\ \vdots \\ {\tilde{\mu }}_{N}{}^{2} \end{array} \end{array}\right],\kappa_{1}=\left[\begin{array}{l} {\tilde{\nu }}_{11}{}^{2}-{\tilde{\mu }}_{1}{}^{2}{\left\|a_{1}\right\|}^{2}\\ \vdots \\ {\tilde{\nu }}_{N1}{}^{2}-{\tilde{\mu }}_{N}{}^{2}{\left\|a_{N}\right\|}^{2} \end{array}\right],\kappa_{2}=\left[\begin{array}{l} {\tilde{\nu }}_{12}{}^{2}-{\tilde{\mu }}_{1}{}^{2}{\left\|a_{1}\right\|}^{2}\\ \vdots \\ {\tilde{\nu }}_{N2}{}^{2}-{\tilde{\mu }}_{N}{}^{2}{\left\|a_{N}\right\|}^{2} \end{array}\right],D=\left[\begin{array}{cc} \begin{array}{l} I_{3}\\ 0_{1\times 3} \end{array} & \begin{array}{l} 0_{3\times 1}\\ 0 \end{array} \end{array}\right],f=\left[\begin{array}{l} 0_{3\times 1}\\ -\frac{1}{2} \end{array}\right],$$

wherein I, 0, and **1** represent identity matrix, zeros matrix, and ones matrix, respectively.

**Definition** **1.** *Suppose* $q:{\mathbb{R}}^{n}\to \mathbb{R}$ *and* $u:{\mathbb{R}}^{n}\to \mathbb{R}$ *to be quadratics and assume* $\left\{\tau \in {\mathbb{R}}^{n}:u\left(\tau \right)=0\right\}$ *is not empty. If*

(16) $$m\neq 0,m^{T}Um=0\Rightarrow m^{T}Qm>0,$$

*where* $Q=\nabla^{2}q$ *,* $U=\nabla^{2}u$ *, then the optimization problem* min{q(τ):u(τ)=0} *has a global minimizer.*

**Definition** **2.** *Let* $q:{\mathbb{R}}^{n}\to \mathbb{R}$ *and* $u:{\mathbb{R}}^{n}\to \mathbb{R}$ *to be the quadratics, and assume that* $\inf\left\{u\left(\tau \right):\tau \in {\mathbb{R}}^{n}\right\}<0<\sup\left\{u\left(\tau \right):\tau \in {\mathbb{R}}^{n}\right\}$ *with* $\nabla^{2}u\neq 0$ *. A vector* $\tau^{*}$ *is a global minimizer of the problem* min{q(τ):u(τ)=0} *if and only if* $u\left(\tau^{*}\right)=0$ *and there is a multiplier* $\lambda^{*}\in \mathbb{R}$ *such that the Kuhn–Tucker condition*

(17) $$\nabla q\left(\tau^{*}\right)+\lambda^{*}\nabla u\left(\tau^{*}\right)=0$$

*is satisfied with*

(18) $$\nabla^{2}q\left(\tau^{*}\right)+\lambda^{*}\nabla^{2}u\left(\tau^{*}\right)$$

*Positive semidefinite.*

Under Definition 1, we can easily verify that (16) holds for the considered problem in (14). Therefore, a global minimizer of the solution of (14) would be acquired. With Definition 2, an optimal solution $y^{k}$ at the $k^{\mathrm{th}}$ iteration would be obtained if there is a multiplier λ such that the Kuhn–Tucker condition, i.e.,

(19) $$\begin{array}{l} \left({\tilde{℘}}^{T}\omega \tilde{℘}+\lambda^{k}D\right)y={\tilde{℘}}^{T}\omega \tilde{\kappa }-\lambda^{k}f,\\ {\left(y^{k}\right)}^{T}Dy^{k}+2f^{T}y^{k}=0,\\ {\tilde{℘}}^{T}\omega \tilde{℘}+\lambda^{k}D≻0, \end{array}$$

At each iteration, the optimal solution is acquired by

(20) $${\hat{y}}^{k}\left(\lambda \right)={\left({\tilde{℘}}^{T}\omega \tilde{℘}+\lambda D\right)}^{-1}\left({\tilde{℘}}^{T}\omega^{T}\tilde{\kappa }-\lambda f\right),$$

where $\lambda^{k*}=\max\left[-diag\left({\tilde{℘}}^{T}\omega \tilde{℘}\right)/diag\left(D\right),\lambda \right]$ and λ is defined as the solution of the function (21) when φ(λ)=0.

(21) $$\varphi \left(\lambda \right)={\left[{\hat{y}}^{k}\left(\lambda \right)\right]}^{T}D{\hat{y}}^{k}\left(\lambda \right)+2f^{T}{\hat{y}}^{k}\left(\lambda \right)$$

The entire process of the proposed method, AMT, could be expressed as shown below Algorithm 1.

**Algorithm 1**: AMT1: Initiation: anchors’ position, target’s position, k=1, $y_{pre}=0$, Threshold=1e−7
2: Calculate the RSS measurements3: **While**
$k<k_{\max}$
**Do**4: Figure out λ at each iteration according to (21)5: Optimal λ at $k^{\mathrm{th}}$ iteration following $\lambda^{k*}=\max\left[-diag\left({\tilde{℘}}^{T}\omega \tilde{℘}\right)/diag\left(D\right),\lambda \right]$
6: Figure out the optimal ${\hat{y}}^{k}$ at each iteration according to (20)7: **If**
$\left\|y^{k}-y_{pre}\right\|/\left\|y^{k}\right\|<Threshold$
8: Break9: **End If**10: $y_{pre}=y^{k}$
11: k=k+1
12: **End While**

**Figure 4:**
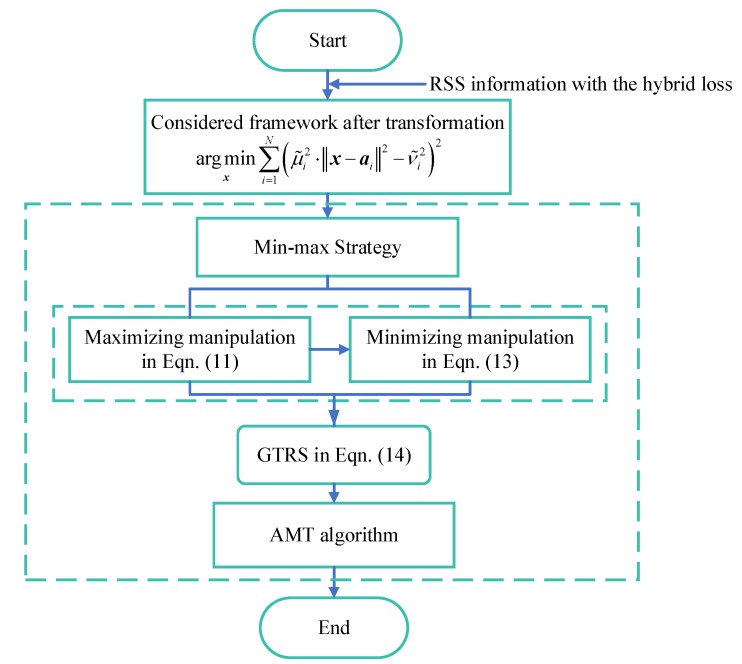
The flowchart of the proposed localization scheme in UWSNs.

In addition, a flowchart is depicted to understand better the proposed localization scheme, referred to as Figure 4.

### 3.3. Cramer–Rao Low Bound (CRLB)

As a covariance matrix representing a lower bound of any unbiased estimators [31], CRLB would be conducted in this part to provide the benchmark. Basically, CRLB could be indicated as the trace of the inverse of the Fisher information matrix (FIM) when the noise is Gaussian, i.e.,

(22) $$CRLB≜\mathrm{Tr}\left(FIM^{-1}\right)=\mathrm{Tr}{\left[\left(\frac{\partial P}{\partial x}\right)\sum^{-1}{\left(\frac{\partial P}{\partial x}\right)}^{T}\right]}^{-1},$$

where ∑ denotes $\mathrm{diag}\left(\sigma_{1},\ldots ,\sigma_{N}\right)$, Tr(⋅) is the trace of a matrix, and

(23) $$\frac{\partial P}{\partial x}=\left(\begin{array}{c} -\psi \frac{x_{1}-a_{1x}}{{\left\|x-a_{1}\right\|}^{2}}-\alpha_{f}\frac{x_{1}-a_{1x}}{\left\|x-a_{1}\right\|},\ldots ,-\psi \frac{x_{1}-a_{Nx}}{{\left\|x-a_{N}\right\|}^{2}}-\alpha_{f}\frac{x_{1}-a_{Nx}}{\left\|x-a_{N}\right\|}\\ -\psi \frac{x_{2}-a_{1y}}{{\left\|x-a_{1}\right\|}^{2}}-\alpha_{f}\frac{x_{2}-a_{1y}}{\left\|x-a_{1}\right\|},\ldots ,-\psi \frac{x_{2}-a_{Ny}}{{\left\|x-a_{N}\right\|}^{2}}-\alpha_{f}\frac{x_{2}-a_{Ny}}{\left\|x-a_{N}\right\|}\\ -\psi \frac{x_{3}-a_{1z}}{{\left\|x-a_{1}\right\|}^{2}}-\alpha_{f}\frac{x_{3}-a_{1z}}{\left\|x-a_{1}\right\|},\ldots ,-\psi \frac{x_{3}-a_{Nz}}{{\left\|x-a_{N}\right\|}^{2}}-\alpha_{f}\frac{x_{3}-a_{Nz}}{\left\|x-a_{N}\right\|} \end{array}\right),$$

with $\psi =\frac{10\alpha }{\ln10}$.

Let

(24) $$\begin{array}{l} \Gamma =\sum_{i=1}^{N}{\left(\psi \frac{x_{1}-a_{ix}}{{\left\|x-a_{i}\right\|}^{2}}+\alpha_{f}\frac{x_{1}-a_{ix}}{\left\|x-a_{i}\right\|}\right)}^{2},ϒ=\sum_{i=1}^{N}\left(\psi \frac{x_{1}-a_{ix}}{{\left\|x-a_{i}\right\|}^{2}}+\alpha_{f}\frac{x_{1}-a_{ix}}{\left\|x-a_{i}\right\|}\right)\cdot \left(\psi \frac{x_{2}-a_{iy}}{{\left\|x-a_{i}\right\|}^{2}}+\alpha_{f}\frac{x_{2}-a_{iy}}{\left\|x-a_{i}\right\|}\right),\\ \Psi =\sum_{i=1}^{N}{\left(\psi \frac{x_{2}-a_{iy}}{{\left\|x-a_{i}\right\|}^{2}}+\alpha_{f}\frac{x_{2}-a_{iy}}{\left\|x-a_{i}\right\|}\right)}^{2},\Xi =\sum_{i=1}^{N}\left(\psi \frac{x_{1}-a_{ix}}{{\left\|x-a_{i}\right\|}^{2}}+\alpha_{f}\frac{x_{1}-a_{ix}}{\left\|x-a_{i}\right\|}\right)\cdot \left(\psi \frac{x_{3}-a_{iz}}{{\left\|x-a_{i}\right\|}^{2}}+\alpha_{f}\frac{x_{3}-a_{iz}}{\left\|x-a_{i}\right\|}\right),\\ \Lambda =\sum_{i=1}^{N}{\left(\psi \frac{x_{3}-a_{iz}}{{\left\|x-a_{i}\right\|}^{2}}+\alpha_{f}\frac{x_{3}-a_{iz}}{\left\|x-a_{i}\right\|}\right)}^{2},\Pi =\sum_{i=1}^{N}\left(\psi \frac{x_{2}-a_{iy}}{{\left\|x-a_{i}\right\|}^{2}}+\alpha_{f}\frac{x_{2}-a_{iy}}{\left\|x-a_{i}\right\|}\right)\cdot \left(\psi \frac{x_{3}-a_{iz}}{{\left\|x-a_{i}\right\|}^{2}}+\alpha_{f}\frac{x_{3}-a_{iz}}{\left\|x-a_{i}\right\|}\right), \end{array}$$

then the FIM could be expressed as

(25) $$FIM=\left[\begin{array}{ccc} \Gamma & ϒ & \Xi \\ ϒ & \Psi & \Pi \\ \Xi & \Pi & \Lambda \end{array}\right]$$

Assume $\left\|\hat{x}-x\right\|=error$, the root mean square error (RMSE) is related to the obtained CRLB through

(26) $$\sqrt{E\left(error^{2}\right)}\geq \sqrt{Tr\left(FIM^{-1}\right)}≜\sqrt{CRLB}$$

### 3.4. Complexity Analysis

Several state-of-the-art methods are discussed for the comparison in terms of the complexity in this part, i.e., weighted least square (WLS) with RSS-only in the non-cooperative scheme in [20], NWLS-K in [24], active set method (ASM) in [8], RNLA in [14], and unconstrained squared range majorization-minimization (USRMM) in [21]. It is noteworthy that the acquirement of an estimate of the target’s location via AMT is by the bisection principle. Therefore, the computational complexity is linear to N, i.e., *O (k_max_·N)*, which is the same as NWLS-K and RNLA when it comes to the maximum iteration $k_{\max}$ (the worst case). It is also worth mentioning that a majorization-minimization method is involved in USRMM; the computation complexity comes to *O (N + k_max_)*. The computational complexity of the rest is concluded in Table 1.

## 4. Numerical Simulations

In this section, a set of numerical simulations are carried out in Matlab to assess the proposed method, compared with WLS [20], NWLS-K [24], ASM [8], USRMM [21], RNLA [14], and CRLB conducted in (26) in different scenarios. It should be noted that the positions of anchors and the target are not fixed due to the dynamics of the currents. In this case, to simulate such a situation, the target and anchor nodes are deployed randomly for each MCT. The area of interest in the simulation is a cube with side length Side. At each MCT, the position of anchors and the target could be expressed as $a_{i}=rand\left(3,1\right)*Side$ and x=rand(3,1)*Side, respectively. The rest of the fixed parameters are concluded in Table 2. In addition, as the calibration of the performance, the root means squared error (RMSE) would be conducted as

(27) $$RMSE=\sqrt{\frac{1}{MCT}\sum_{mct=1}^{MCT}\left\|{\hat{x}}_{mct}-x_{mct}\right\|},$$

where $x_{mct}$ and ${\hat{x}}_{mct}$ denote the exact position and the estimate in the $MCT^{\mathrm{th}}$ trial.

### 4.1. Scenario with Variable $\alpha_{f}$

**Figure 5:**
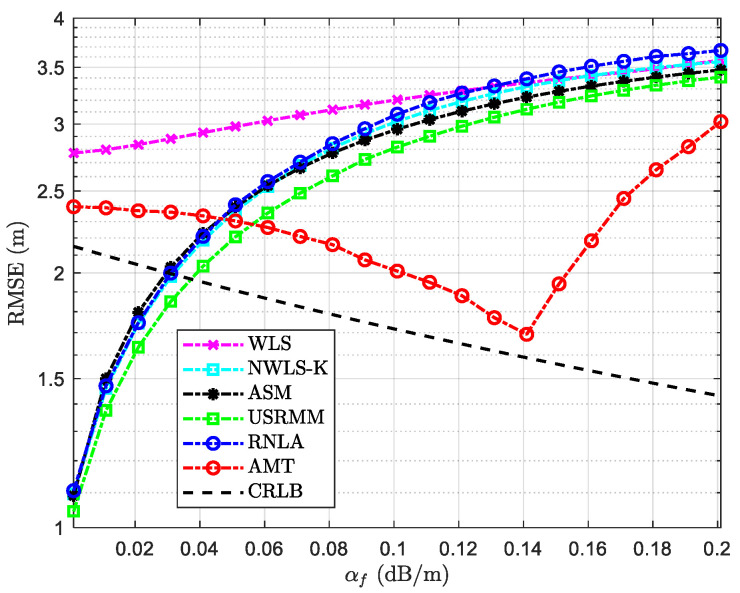
RMSE versus variable $\alpha_{f}$ with $P_{0}=-55\;\mathrm{dBm}$, N=10, $\sigma_{i}^{2}=4\;\mathrm{dB}$, and the side length of the cube Side=50 m.

The RMSE versus variable $\alpha_{f}$ is depicted in Figure 5. In addition to the fixed parameters in Table 2, the rest of the parameters in the scenario with variable $\alpha_{f}$ are shown in Table 3. It is noteworthy that the frequency that the acoustic system operates varies from 10 kHz to 1000 kHz [26], where the corresponding $\alpha_{f}$ could be 0.001 dB/m to 0.32 dB/m [27]. However, we only conduct the simulation with $\alpha_{f}$ varying from 0.001 dB/m to 0.2 dB/m, which is more practical for UWSNs to operate [13]. In addition, it should be noted that the maximum of the absorption could be known because the side length of the cube is determined according to Figure 3 if Side=50 m, i.e., $c_{\max}=50\sqrt{5}\alpha_{f}\;\mathrm{dB}$. Theoretically, the increase of $\alpha_{f}$ accelerate the signal attenuation caused by the absorption, according to Equation (1). Therefore, it can be seen from Figure 5 that the performance of the methods, including WLS, NWLS-K, ASM, USRMM, and RNLA, deteriorates over the rise in $\alpha_{f}$. On the contrary, the performance of the proposed method (AMT) is inverse proportion to the rise in $\alpha_{f}$ from 0.001 dB/m to 0.14 dB/m, and get close to the trend of CRLB. The outperformance of AMT can be explained to some extent by the fact that we mitigate the worst case of the absorption via a min–max strategy in the iteration. Nevertheless, it should be noted that if the bias exceeds the tolerance of the estimator, the performance of mitigation would degrade due to the limits of the bisection method. In other words, the performance of AMT would deteriorate if the absorption loss reaches a tolerant value. As shown in Figure 5, the tolerance is $\alpha_{f}=0.14\;\mathrm{dB}/m$. In addition, we can observe that when $\alpha_{f}<0.06\;\mathrm{dB}/m$, the error of AMT is more significant than most of the methods due to the intrinsic error of the estimator. When $\alpha_{f}$ is relatively low, the absorption loss for AMT is relatively larger than the rest because we assume the worst situation (maximum distance between nodes). Fortunately, the error could be reduced when $\alpha_{f}$ increases via iteration. Similarly, the same situation comes to CRLB when $\alpha_{f}<0.04\;\mathrm{dB}/m$. The localization accuracy of CRLB is relatively lower than that of some considered methods due to the intrinsic error when $\alpha_{f}$ is low. Although the rate of deterioration of AMT is larger than the rest when $\alpha_{f}>0.14\;\mathrm{dB}/m$, the performance of AMT is better than the rest.

### 4.2. Scenario with Variable $\sigma_{i}^{2}$

**Figure 6:**
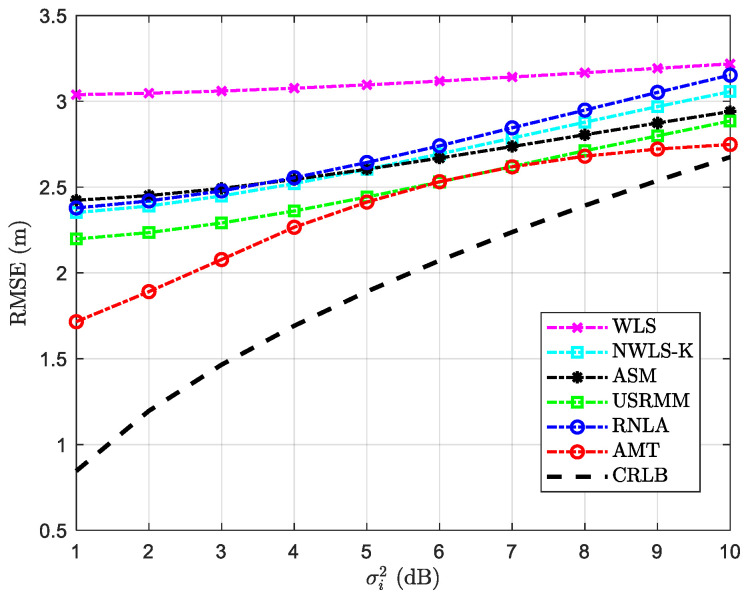
RMSE versus variable $\sigma_{i}^{2}$ with $P_{0}=-55\;\mathrm{dBm}$, N=10, $\alpha_{f}=0.06\;\mathrm{dB}/m$, and the side length of the cube Side=50 m.

The result of the RMSE versus variable $\sigma_{i}^{2}$ is shown in Figure 6. In addition to the fixed parameters in Table 2, the rest of the parameters in the scenario with variable $\sigma_{i}^{2}$ are shown in Table 4. As expected, the RMSE increases as $\sigma_{i}^{2}$ grows, among which the performance of WLS is the poorest, and RNLA seems to be more sensitive to the growing of $\sigma_{i}^{2}$. The ratio of deterioration for RNLA is the largest than that of the considered methods. From Figure 6, we could see that AMT beats the others and gets close to CRLB. When $\sigma_{i}^{2}$ is relatively low, the performance of AMT seems to be better than the others. Even though similar results are performed between AMT and USRMM, especially when $\sigma_{i}^{2}=6\;\mathrm{and}\;7\;\mathrm{dB}$, the margin is more sizeable since $\sigma_{i}^{2}$ increases further from 7 dB.

### 4.3. Scenario with Variable N

**Figure 7:**
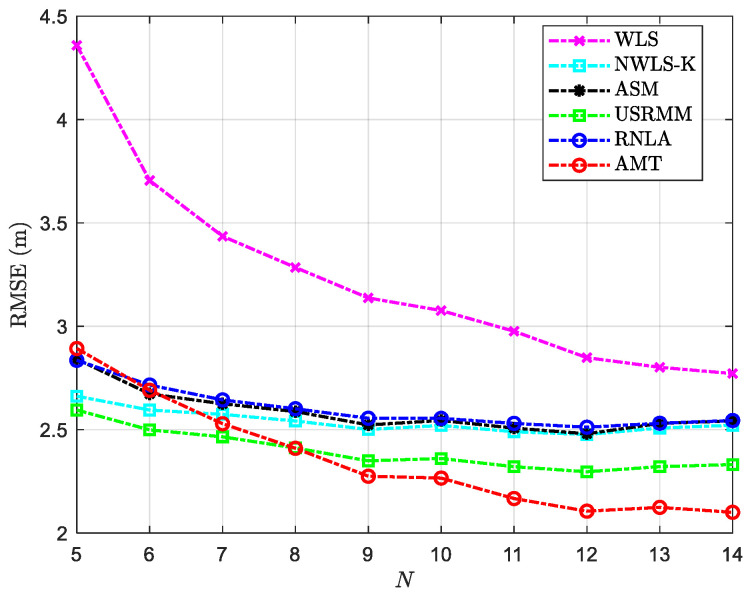
RMSE versus variable N with $P_{0}=-55\;\mathrm{dBm}$, $\sigma_{i}^{2}=4\;\mathrm{dB}$, $\alpha_{f}=0.06\;\mathrm{dB}/m$, and the side length of the cube Side=50 m.

The deviation of the considered methods under variable N is shown in Figure 7. In addition to the fixed parameters in Table 2, the rest of the parameters in the scenario with variable N are shown in Table 5. It should be mentioned that the available information for localization increases while N grows. Thus, the performance of the methods is improved when the number of anchors increases to 14 from 5. From Figure 7, we could see that the number of anchors matters the most to WLS, where the localization accuracy increases by 36%, compared with that of 27% for AMT, 11% for ASM, 10% for RNLA, 10% for USRMM, and 5% for NWLS-K. Among the considered methods, the performance of AMT is relatively satisfactory compared with the others, albeit the error of AMT is equal to or greater than that of most of the methods when N=5 and 6. The outperformance seems to be remarkable when N=14. It should be noted that the more anchors engaged in the localization, the more available measurement information can be used. In other words, the more, the better. However, from the practical point of view, the extra expense would increase over the rise in N. In this case, as for a relatively small area of interest, for instance, the shallow water with depth within 100 m, the number of anchors is generally from 8 to 20 [24]. From Figure 7, the proposed method seems to be the better choice when it comes to practice.

### 4.4. Scenario with Variable $P_{0}$

**Figure 8:**
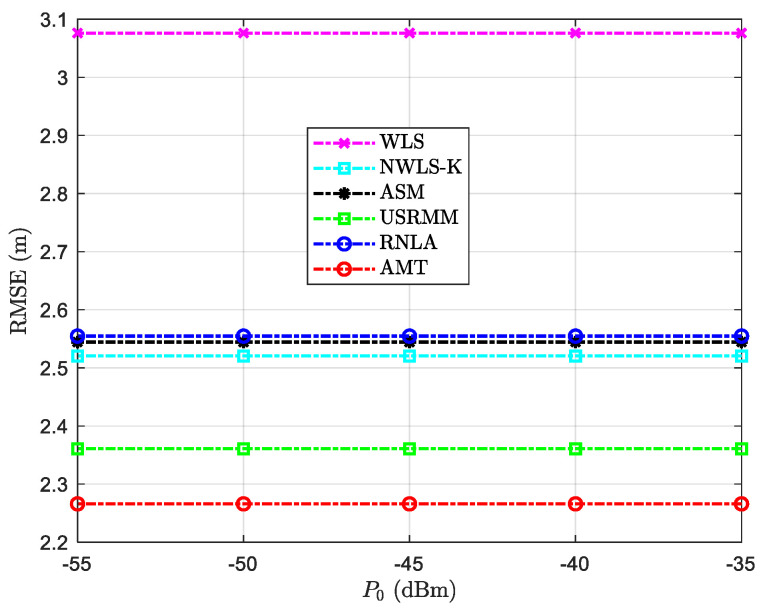
RMSE versus variable $P_{0}$ with N=10, $\sigma_{i}^{2}=4\;\mathrm{dB}$, $\alpha_{f}=0.06\;\mathrm{dB}/m$, and the side length of the cube Side=50 m.

The RMSE versus variable $P_{0}$ is depicted in Figure 8. In addition to the fixed parameters in Table 2, the rest of the parameters in the scenario with variable $P_{0}$ are shown in Table 6. It can be seen that the considered algorithm is robust to $P_{0}$, and the performance has relative stability. From Figure 8, we could see that similar results are performed for RNLA, ASM, and NWLS-K, of which the localization error is around 2.5 m compared with that of 3.1 m for WLS. It is obvious that the deviation of AMT is the lowest among them, albeit the difference between AMT and USRMM is small (almost 0.1 m).

### 4.5. Scenario with the Variable Side Length of the Cube

**Figure 9:**
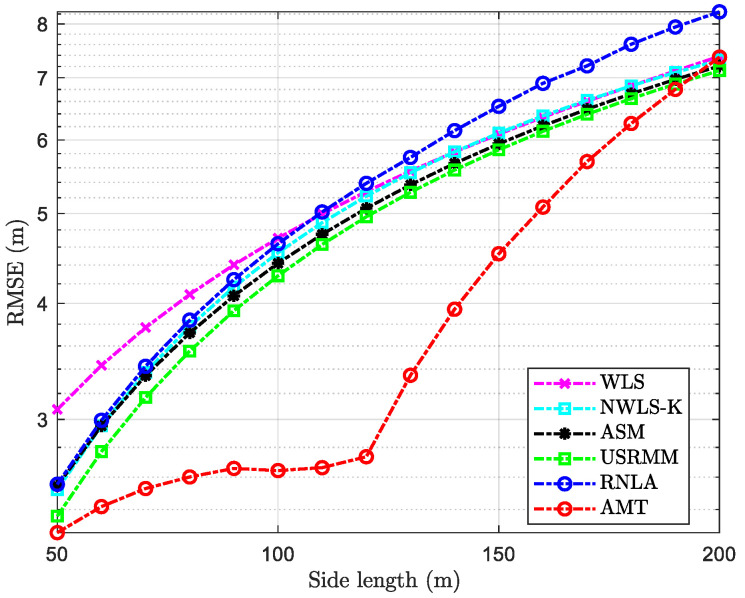
RMSE versus variable side length with N=10, $\sigma_{i}^{2}=4\;\mathrm{dB}$, $\alpha_{f}=0.06\;\mathrm{dB}/m$, and $P_{0}=-55\;\mathrm{dBm}$.

It is essential to conduct the simulation in different side lengths of the area because the absorption bias is related to the maximum distance between anchors and the target in terms of AMT. In addition to the fixed parameters in Table 2, the rest of the parameters in the scenario with variable Side are shown in Table 7. As shown in Figure 9, the side length varies from 50 m to 200 m, which means the range of $c_{\max}$ is from $50\alpha_{f}\sqrt{5}\;\mathrm{dB}$ to $200\alpha_{f}\sqrt{5}\;\mathrm{dB}$, according to Figure 3. As a result that the distance between anchors and the target is in proportion to the side length, the adverse impact of the absorption on localization accuracy would increase over the rise in the side length. Thus, the performance of the considered methods degrades while the side length increases to 200 m from 50 m. Interestingly, the ratio of deterioration is relatively low for AMT when the side length is less than or equal to 120 m. It indicates that AMT could, to some extent, mitigate the adverse effect of absorption on localization. However, the ratio of deterioration climbs dramatically when the side length increases further from 120 m, compared with the others. Nevertheless, the performance of AMT is better than others when the side length is less than 200 m, albeit a relatively large ratio of deterioration. From the results of Figure 9, it seems that the proposed method, AMT, could be adopted when the UWSNs are deployed in shallow water. When it comes to the deep sea, AMT is not a preferable method for localization due to the exponential increase of the signal attenuation and the absorption.

### 4.6. Cumulative Distribution Function (CDF)

**Figure 10:**
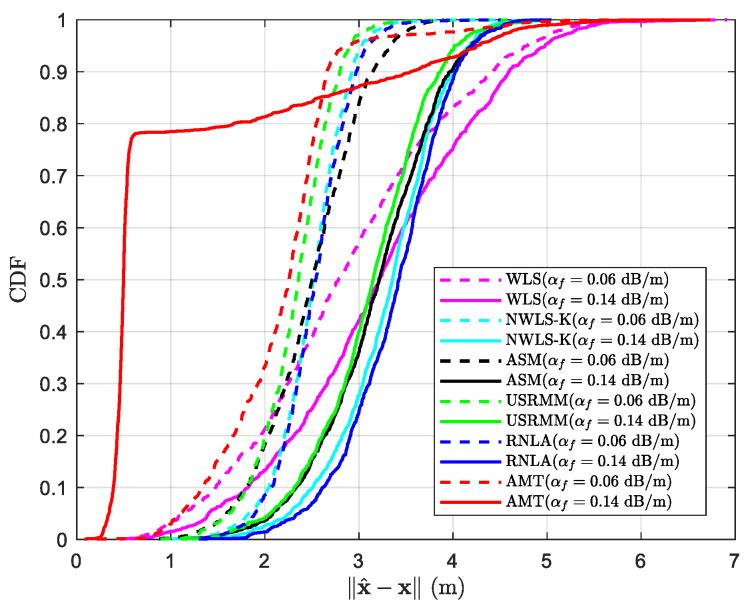
CDF of $\left\|\hat{x}-x\right\|$ with N=10, $\sigma_{i}^{2}=4\;\mathrm{dB}$, $P_{0}=-55\;\mathrm{dBm}$, Side=50 m, and $\alpha_{f}=0.06\;\mathrm{dB}/m\;\mathrm{or}\;0.14\;\mathrm{dB}/m$.

Figure 10 shows the cumulative distribution function (CDF) of $\left\|\hat{x}-x\right\|$ for different algorithms when $\alpha_{f}=0.06\;\mathrm{dB}/m\;\mathrm{and}\;0.14\;\mathrm{dB}/m$, respectively. In addition to the fixed parameters in Table 2, the rest of parameters are shown in Table 8. From Figure 10, we could see that AMT achieves $\left\|\hat{x}-x\right\|=2.54\;m$ at almost 80% when $\alpha_{f}=0.06\;\mathrm{dB}/m$, whereas USRMM, NWLS-K, RNLA, ASM, and WLS achieve $\left\|\hat{x}-x\right\|=2.64\;m$, $\left\|\hat{x}-x\right\|=2.78\;m$, $\left\|\hat{x}-x\right\|=2.83\;m$, $\left\|\hat{x}-x\right\|=2.93\;m$, and $\left\|\hat{x}-x\right\|=3.84\;m$ at the same probability, respectively. The situation gets worse when it comes to $\alpha_{f}=0.14\;\mathrm{dB}/m$, where, except for AMT, the methods achieve the same probability at more significant error than that of $\alpha_{f}=0.06\;\mathrm{dB}/m$, i.e., $\left\|\hat{x}-x\right\|=3.61\;m$ for USRMM, $\left\|\hat{x}-x\right\|=3.75\;m$ for NWLS-K, $\left\|\hat{x}-x\right\|=3.83\;m$ for RNLA, $\left\|\hat{x}-x\right\|=3.72\;m$ for ASM, and $\left\|\hat{x}-x\right\|=4.17\;m$ for WLS. Regarding AMT, the adverse impact of the absorption seems to be mitigated in a way due to the min–max strategy that we minimized the worst situation, and the performance is improved to $\left\|\hat{x}-x\right\|=1.66\;m$ at almost 80%.

### 4.7. Computational Time

**Figure 11:**
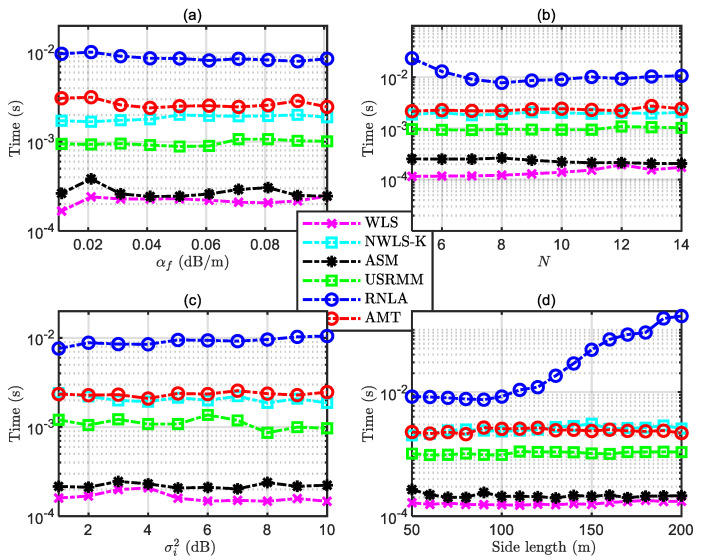
Computational time comparisons under different conditions: (**a**) Computational time under variable $\alpha_{f}$ with $P_{0}=-55\;\mathrm{dBm}$, N=10, $\sigma_{i}^{2}=4\;\mathrm{dB}$, and the side length of the cube Side=50 m, (**b**) computational time under variable N with $P_{0}=-55\;\mathrm{dBm}$, $\sigma_{i}^{2}=4\;\mathrm{dB}$, $\alpha_{f}=0.06\;\mathrm{dB}/m$, and the side length of the cube Side=50 m, (**c**) computational time under variable $\sigma_{i}^{2}$ with $P_{0}=-55\;\mathrm{dBm}$, N=10, $\alpha_{f}=0.06\;\mathrm{dB}/m$, and the side length of the cube Side=50 m, and (**d**) computational time under variable side length with N=10, $\sigma_{i}^{2}=4\;\mathrm{dB}$, $\alpha_{f}=0.06\;\mathrm{dB}/m$, and $P_{0}=-55\;\mathrm{dBm}$.

In addition to the RMSE, the computational time is another crucial factor for an estimator, which could intuitively reflect the efficiency of an algorithm. In this context, the simulation with subject to the computational time is carried out in different scenarios, and the results are depicted in Figure 11. The corresponding parameters in Figure 11 are (1) referred to in Table 2 and Table 3 for Figure 11a; (2) referred to in Table 2 and Table 5 for Figure 11b; (3) referred to in Table 2 and Table 4 for Figure 11c; and (4) referred to in Table 2 and Table 7 for Figure 11d. We could see that the time consumption of RNLA is much more than others. This is because a block prox-linear, the method to figure out the global solution, involves RNLA, which needs extra time for searching. Regarding the time consumption of AMT, the performance is not remarkable but acceptable, compared to that of RMSE. The average time consumption of AMT is around 2.7 × 10^−3^ s for each MCT, which is similar to that of NWLS-K but a little bit more than USRMM, ASM, and WLS.

## 5. Conclusions

In this paper, an absorption mitigation technique, namely AMT, is proposed to mitigate the negative influence of the absorption on localization in UWSNs. The considered localization problem is reshaped to a GTRS framework via a set of tight approximations for small noise powers. In addition, a min–max strategy is presented to minimize the worst situation for the absorption, wherein the problem is divided into two subproblems and jointly solved by a bisection method. The simulations confirm the effectiveness of the proposed algorithm in different scenarios compared with the state-of-the-art approaches. The results reveal that the proposed method, AMT, seems to more suitable for localization in the UWSNs deployed in the shallow water. Additionally, it should be noted that if the absorption loss exceeds the tolerance of AMT, the performance of mitigation would degrade, referred to in Figure 5 and Figure 9. After carrying out plenty of simulations, we find it that the tolerant absorption loss of AMT is around 16 dB as the area of interest is a cube.

## Figures and Tables

**Table 1:** Complexity analysis of the considered methods.

Method	Complexity
WLS	*O (N)*
NWLS-K	*O (k_max_·N)*
ASM	*O (N)*
USRMM	*O (N + k_max_)*
RNLA	*O (k_max_·N)*
AMT	*O (k_max_·N)*

**Table 2:** Some fixed parameters in simulations.

Parameters	Value
α (path loss exponent)	3
MCT (Monte Carlo Trials)	1000
$k_{\max}$ (Maximum iteration)	1000
$d_{0}$ (Reference distance)	1 m

**Table 3:** Parameters in the scenario with variable $\alpha_{f}$.

Parameters	Value
$P_{0}$ (Transmit power)	−55 dBm
N (The number of anchors)	10
$\sigma_{i}^{2}$ (Variance)	4 dB
Side (Side length of the area)	50 m

**Table 4:** Parameters in the scenario with variable $\sigma_{i}^{2}$.

Parameters	Value
$P_{0}$ (Transmit power)	−55 dBm
N (The number of anchors)	10
$\alpha_{f}$ (Absorption coefficient)	0.06 dB/m
Side (Side length of the area)	50 m

**Table 5:** Parameters in the scenario with variable N.

Parameters	Value
$P_{0}$(Transmit power)	−55 dBm
$\sigma_{i}^{2}$(Variance)	4 dB
$\alpha_{f}$(Absorption coefficient)	0.06 dB/m
Side(Side length of the area)	50 m

**Table 6:** Parameters in the scenario with variable $P_{0}$

Parameters	Value
N (The number of anchors)	10
$\sigma_{i}^{2}$ (Variance)	4 dB
$\alpha_{f}$ (Absorption coefficient)	0.06 dB/m
Side (Side length of the area)	50 m

**Table 7:** Parameters in the scenario with variable Side

Parameters	Value
N (The number of anchors)	10
$\sigma_{i}^{2}$ (Variance)	4 dB
$\alpha_{f}$ (Absorption coefficient)	0.06 dB/m
$P_{0}$ (Transmit power)	−55 dBm

**Table 8:** Parameters in the comparison of cumulative distribution function (CDF) of $\left\|\hat{x}-x\right\|$.

Parameters	Value
N (The number of anchors)	10
Side (Side length of the area)	50 m
$\sigma_{i}^{2}$ (Variance)	4 dB
$\alpha_{f}$ (Absorption coefficient)	0.06 dB/m or 0.14 dB/m
$P_{0}$ (Transmit power)	−55 dBm
